# Antigenic “Hot- Spots” on the TSH Receptor Hinge Region

**DOI:** 10.3389/fendo.2018.00765

**Published:** 2019-01-07

**Authors:** Simeng Sun, Sarawut Summachiwakij, Ora Schneck, Syed A. Morshed, Risheng Ma, Rauf Latif, Terry F. Davies

**Affiliations:** Thyroid Research Unit, Department of Medicine, James J. Peters VA Medical Center, Icahn School of Medicine at Mount Sinai, New York, NY, United States

**Keywords:** TSH receptor, hinge antibodies, neutral antibodies, ectdomain, Graves' disease (GD)

## Abstract

The TSH receptor (TSHR) hinge region was previously considered an inert scaffold connecting the leucine-rich ectodomain to the transmembrane region of the receptor. However, mutation studies have established the hinge region to be an extended hormone-binding site in addition to containing a region which is cleaved thus dividing the receptor into $\overset{’}{\underset{|}{\alpha }}$ (A) and β (B) subunits. Furthermore, we have shown *in-vitro* that monoclonal antibodies directed to the cleaved part of the hinge region (often termed “neutral” antibodies) can induce thyroid cell apoptosis in the absence of cyclic AMP signaling. The demonstration of neutral antibodies in patients with Graves' disease suggests their potential involvement in disease pathology thus making the hinge a potentially important antigenic target. Here we examine the evolution of the antibody immune response to the entire TSHR hinge region (aa280–410) after intense immunization with full-length TSHR cDNA in a mouse (BALB/c) model in order to examine the immunogenicity of this critical receptor structure. We found that TSHR hinge region antibodies were detected in 95% of the immunized mice. The antibody responses were largely restricted to residues 352–410 covering three major epitopes and not merely confined to the cleaved portion. These data indicated the presence of novel antigenic “hotspots” within the carboxyl terminus of the hinge region and demonstrate that the hinge region of the TSHR contains an immunogenic pocket that is involved in the highly heterogeneous immune response to the TSHR. The presence of such TSHR antibodies suggests that they may play an active role in the immune repertoire marshaled against the TSHR and may influence the Graves' disease phenotype.

## Introduction

The thyroid stimulating hormone receptor (TSHR) is the major regulator of thyroid gland function and also a major autoantigen in Graves' disease (GD) which is caused by stimulating TSHR autoantibodies inducing thyroid gland hyperactivity (1, 2). Autoantibodies to the TSHR found in patients with Graves' disease may be stimulating, blocking, or neutral based on their modulation of cyclic AMP signaling (3). All stimulating and most blocking receptor antibodies bind conformationally to the leucine-rich repeat domain (LRD), part of the large ectodomain (ECD) of the TSHR while neutral antibodies recognize linear epitopes largely within the hinge region of the receptor which links the LRD to the transmembrane (TM) sequences. The stimulating TSHR antibodies, like TSH itself, activate the TSHR on the basolateral surface of thyroid epithelial cells leading to multiple G protein activation–predominantly Gαs (4, 5) but also Gαq-and Gβγ signaling pathways (5, 6), while blocking antibodies inhibit TSH action on the receptor by physically preventing the interaction of TSH with the LRD (7). Crystallization of the TSHR ECD bound to human stimulating and blocking monoclonal antibodies to the TSHR has provided insight into antibody contact residues showing the LRD as the major binding region (8, 9). Although the crystal structures did not include the full hinge region we have provided evidence that TSHR-Abs also bind to this area (10). The crystal structure of the hinge region of the FSHR (11) has provided evidence of the structural features of this region which likely applies to all glycoprotein hormone receptors including the TSHR and has allowed homology modeling of the entire ectodomain (12) and thus extended our understanding on ligand displacement of specific hinge fragments.

Previously, the hinge region of the TSHR, which spans 114 amino acids, was considered as an inert scaffold connecting the LRD to the TM region of the receptor (13) although some later studies have defined this region to extend from residues 289–409 (14) and even from aa 280–410 (15). However, studies using monoclonal antibodies and synthetic peptides have demonstrated that certain epitopes within the hinge region are involved in TSH binding, and mutation studies have established the hinge region to be an extended hormone-binding site (16). The hinge region also encompasses a unique 50 amino acid cleaved region (amino acids 316–366). Antibodies directed to the hinge region are often termed “neutral” antibodies, even though studies from our laboratory have established that they are not neutral in function (17, 18). Our studies have also shown that hinge region specific neutral antibodies might also play a role in inhibiting the receptor from being cleaved and released from the cell surface possibly by dimerizing two receptors (19). Furthermore, we have shown both *in vitro* and *in vivo* that neutral antibodies have the ability to lead to cell stress and apoptosis of thyrocytes, which appears to only, happen in the absence of cyclic AMP signaling (20, 21). Identification of “neutral” TSHR antibodies in patients with Graves' disease, therefore, suggests their potential involvement in the disease process (18).

Realizing the important role of the hinge region of the TSHR in structure-function (15), we set out to evaluate the antigenicity of this entire region using a recently established intense cDNA immunization scheme (22). The hinge region of the TSHR is theoretically peppered with linear epitopes and the present study showed that the TSHR immunized mice almost all developed antibodies to the hinge region but predominantly to three antigenic peptides encompassing the carboxyl terminus of the hinge indicating the existence of antigen “hot spots.”

## Materials and Methods

### Animals

Female BALB/c mice, ages between 6 and 8 weeks, were purchased from Jackson Labs (*n* = 30). All animal studies were approved by the Institutional Animal Care and Use Committee (IACUC) and performed with all standard animal care precautions in a pathogen-free facility.

### Immunization

**Figure 1:**
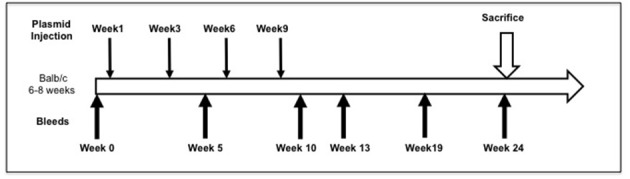
Immunization scheme of the mouse model. Female Balb/c mice of 6–8 weeks were used in this study. Test animals (*n* = 20) were injected with expression plasmids pEGFP-hTSHR and control animals (*n* = 10) with pEGFP-N1 empty vector, respectively with 50 μl of plasmid DNA in the bicep femoris muscle and electroporated as described in materials and method. This immunization scheme was done every 3 weeks for a total of 4 times. The mice were bled at 0, 5, 10, 13, 19, and 24 weeks.

Full-length pEGFP-hTSHR described previously (23) and empty vector pEFGP-N1 plasmid DNA was prepared using QIAGEN plasmid mega kit (Cat # 12181). After spectrophotometric measurement of the purified DNA, it was diluted to 1 mg/mL in calcium and magnesium free 1X PBS prior to immunization. The animals were anesthetized and injected with 50 μL of plasmid DNA (1 mg/mL) into the biceps femoris muscle. The injected DNA was electroporated using the ECM830 square wave electroporator (BTX) with 7 mm caliper electrodes at 200 V/cm^2^. The current was applied in 10–20 ms^2^ wave pulses at 1 Hz. This muscle immunization was performed four times at 3-week intervals. Blood was collected by submandibular bleeding at weeks 5, 10, 13, 19, and 24 (Figure 1). Serum samples were separated and stored at −20°C.

### Thyroid Function Testing

Serum samples were diluted 1:10 in assay buffer and measured for T4 and TSH levels using the Millipore MAP thyroid magnetic bead method (Cat # RTHYMAG-30 K) as per the manufacturer's protocol. The cut-off was specified by ±2 standard deviations from the average of the control empty vector immunized samples.

### TSH Bioassay

Luciferase assays for TSH-like action were performed as described previously (24). Briefly, a 384-well plate was seeded with 15,000 cells/well of CHO-TSHR cells co-expressing a CRE-luciferase (TSHR-Glo) construct. Thirty microliters of purified serum IgG at a concentration of 1 μg/mL or serum diluted 1:10 to 1:100 per well was used in the stimulation assay or inhibition assay for blocking antibodies. All sera or IgG were diluted in F12 medium and incubated with TSHR-Glo cells for 4 h prior to addition of luciferase substrate. For the inhibition assays, the diluted samples (1:100) were added to the wells first, and after 60 min of pre-incubation, the wells were spiked with bovine TSH (final concentration of 50 μU/well) followed by 4 h of incubation at 37°C in a CO_2_ incubator with >85% humidity prior measuring luminescence in the ClarioStar microplate reader (BMG Inc). Positive and negative controls used were bovine TSH/ medium alone and sera diluted from control vector immunized mice.

### TSHR Antibodies Detected by Flow-Cytometry

CHO-TSHR expressing cells were used for the detection of TSHR antibodies. These cells were grown overnight at 37**°**C in F12 medium containing 100 units of penicillin and streptomycin and 10% FBS. The cells from the plate were detached using 1 mM EGTA/EDTA and washed twice with 1X PBS, filtered using 75 μM filter and suspended in 100 μL of FACS staining buffer (1X PBS with 0.2% sodium azide and 2% FBS) at a density of 1 × 10^6^ cells/tube. Test and control serum diluted 1:50 were incubated with cells for an hour at room temperature and TSHR receptor binding antibodies were detected using anti-mouse antibody Fab' phycoerthrin (PE) labeled secondary antibody. Tube with no primary antibody and anti TSHR mAb RSR1 (0.1 μg/mL) were used as negative and positive controls, respectively in this unfixed FACS assay. The results were expressed as the percentage positive cells detected in the test samples compared to the control serum samples. All sera were tested at 10 and 24 weeks after immunization for these antibodies.

### Detection of TSHR Hinge Region Antibodies

**Figure 2:**
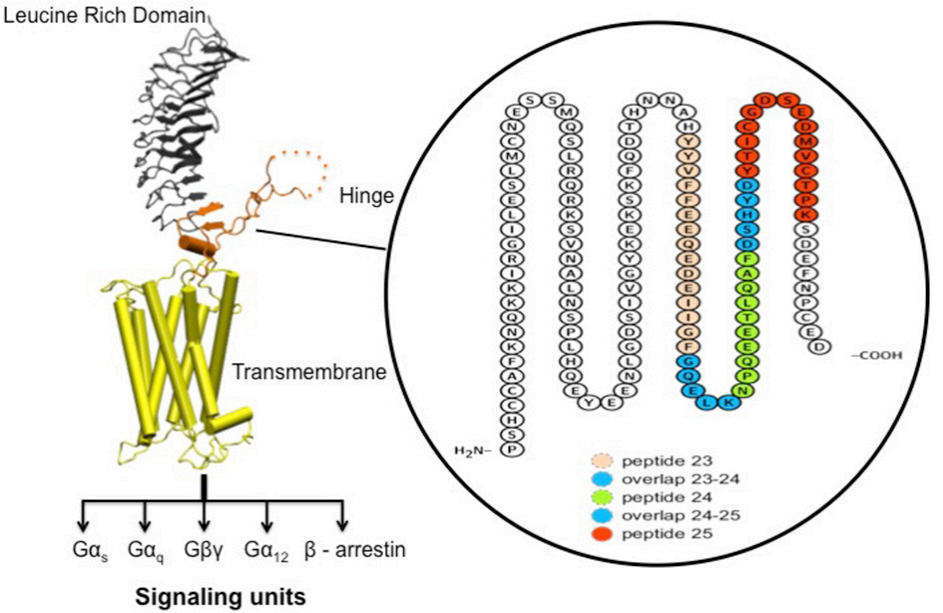
Receptor structure and hinge region amino acid sequence. **(Left)** Model of the full-length human TSH receptor showing the major concave leucine-rich domain (gray), the large hinge region (orange) connecting to the helical transmembrane domain (yellow) with its short carboxyl tail and extra- and intra- cellular loops (25). The intracellular loops of receptor harbor sites for downstream effectors such Gαs, Gαq, Gβγ, Gα12, and recruitment of β-arrestins thus forming the array of signaling units of the TSHR. **(Right)** A magnified image with amino acid sequence of the hinge region (aa 280–410) of the receptor with the “hot-spot” peptides marked in color. Shown here are the 3 “hot-spots” of the hinge region described in this study: peptide 23 (residues 352–371), peptide 24 (residues 367–386), and peptide 25 (residues 382–401) with there overlapping regions indicated in blue here. Note the 50 amino acid cleaved region (amino acids 316–366- indicated as dotted line in the above model) of the hinge has an overlap with peptide 23.

In a series of qualitative ELISA's we used 9 overlapping 20-mer peptides spanning the entire human TSHR hinge region (amino acids 280–410) as indicated schematically in Figure 2. Briefly, the peptide ELISA were performed by coating 100 μL/well of peptides dissolved in bicarbonate buffer pH 9.6 at 10 μg/mL. The plates were blocked with 2% BSA for 2 h at 25**°**C. Mice sera were diluted 1:100 and incubated for 1 h at 25**°**C. Anti-mouse IgG-Fab' HRP was used as the detection antibody. The optical density (OD) was obtained at 450 nm in a CalrioStar microplate reader (BMG Inc). The ELISA approach proved highly specific when tested with control peptides. The cut-off was specified by ±2 standard deviations from the average of the control serum samples for each of the peptide plates.

### Histology

At the end of 24 weeks, the thyroid glands and heads of the control and test animals were retrieved and fixed in 10% buffered formalin in 0.9% NaCl. After fixation, the thyroid gland was embedded in paraffin and processed further. To examine the retro-orbital tissue of these animals, the heads were decalcified for 2 weeks prior to coronal sectioning along the orbit in order to obtain sections with the optic nerve. Representative slides of thyroid and orbital section from the individual immunized or control animals were stained with hematoxylin and eosin (H&E) to examine the gross histological changes. Images of the stained H & E slides were acquired using a Hamamatzu NanoZoomer Digital slide scanner at 20x magnification. NDP.view 2 viewing software was used for further magnification to examine the tissue for histological changes. Images thus obtained were used for quantification of adipose tissue around the optic nerve in the retro-orbital area by drawing ROIs around the optic nerve and the area occupied by the adipose tissue using ImageJ and adiposity calculated as described previously (22).

### Data Analysis

As required, statistical analysis of data was performed using GraphPad Prism Version 6.04 and two-tailed *t*-tests were performed by the same software. All error bars plotted are *SD* values from at least triplicate measurements.

## Results

### Assessment of the Immunization Model

**Figure 3:**
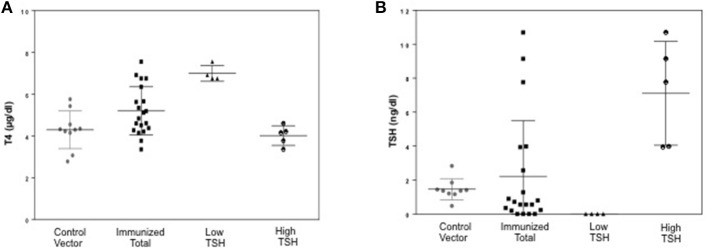
Thyroid function tests of immunized animals. **(A)** Serum TSH levels: T4 and TSH were detected using multiplex bead assay (Millipore) as described in materials and method. The average TSH level was 1.46 ± 1.26 ng/dL in the control animals vs. 2.22 ± 6.56 ng/dL in the immunized group. When the immunized group was subdivided into low and high TSH groups, 25% of mice had high TSH levels. **(B)** Serum T4 levels: Average T4 level was 4.29 ± 1.72 μg/dL in the control animals vs. 5.20 ± 2.32 μg/dL in the immunized group. 20% of the immunized animals had elevated T4 with corresponding suppressed TSH. There was significant difference (*p* < 0.0001) between the high TSH and the control group only.

**Figure 4:**
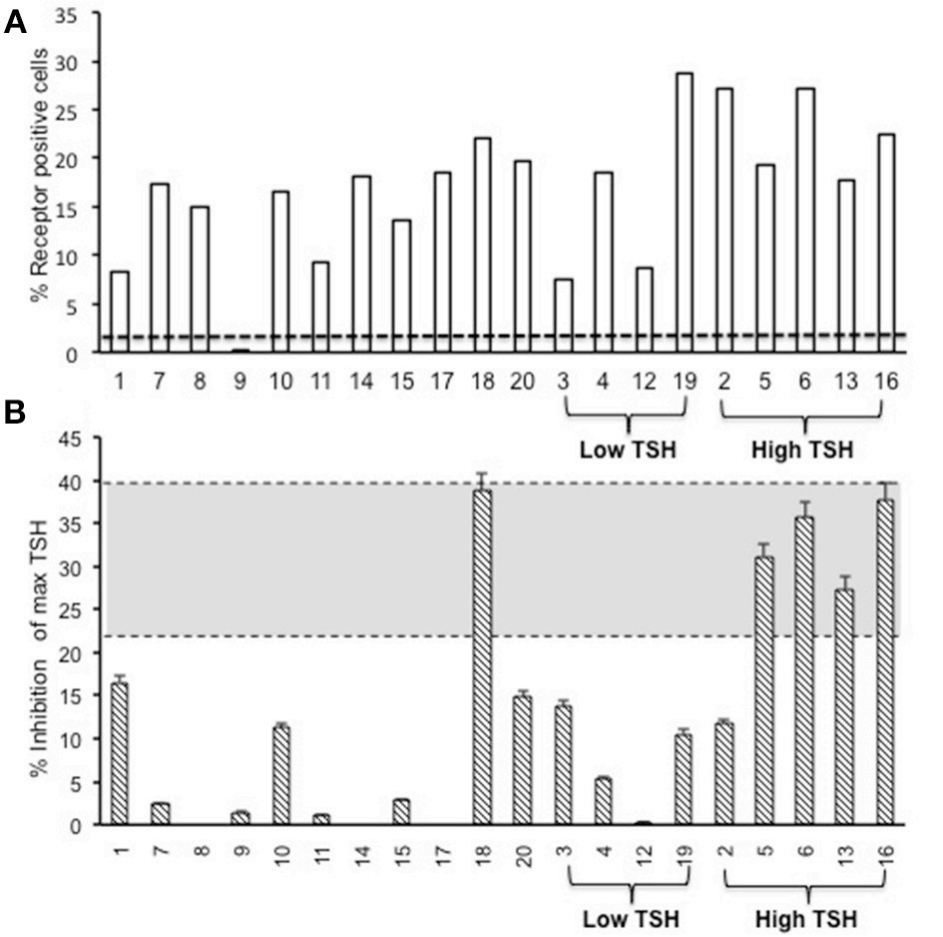
Total TSH receptor antibodies in immunized mice. **(A)** Percentage of TSHR antibody: Flow cytometry was carried out at week 10 with serum samples from immunized and control animals using CHO-TSHR expressing cells to estimate TSH receptor binding antibodies. By this analysis, 95% of the immunized mice had conformational TSHR binding antibodies in their serum. The dotted line is the average response seen in the 10 control animals for TSHR antibodies (≤0.2%). **(B)** TSHR inhibiting antibody. To examine TSH blocking antibodies in the serum of immunized animals we used the TSHRGlo cells and performed the assay as described in M & M. K1-70 blocking monoclonal antibody was used as a positive control which gave a 39.7% inhibition to 60 μU of bTSH in the assay, as indicated by the top dotted line. The lower cutoff (bottom dotted line) is 2 standard deviations above the average of the control mice, which was 20.4%. By these criteria we observed 5 out of the 20 hTSHR immunized animals had blocking antibodies at week 10 and 80% of these were mice with high TSH.

To evaluate the immunogenicity of the human full-length TSHR plasmid compared to the empty vector we immunized BALB/c mice at 3-week intervals and blood samples were collected as schematically shown in Figure 1. At week 10, the T4 level of the control plasmid immunized mice was 4.29 ± 1.8 μg/dL compared to 5.20 ± 2.32 μg/dL in the receptor plasmid immunized animals. Twenty percentage of individual receptor immunized animals had elevated but variable T4 levels with correspondingly suppressed TSH indicating hyperthyroidism (Figures 3A,B). Five out of 20 (25%) receptor immunized mice had significantly elevated TSH indicative of hypothyroidism (Figures 3A,B). However, no TSHR stimulating antibodies were detected in any of the sera from TSHR cDNA immunized mice (data not shown). Five of the 20 immunized mice showed blocking antibodies at week 10 including 4 of the 5 hypothyroid mice with elevated TSH (Figure 4B).

### Antibodies to Full-Length TSHR

The second step in our assessment of the full-length receptor immunization model was the detection of TSHR binding antibodies to human TSHR as stably expressed on CHO cells and assessed by flow-cytometry. At 10 weeks post-immunization 95% of the immunized animals had detectable TSHR antibodies and the relative percentage positivity ranged from 7 to 36% (Figure 4A). The control animals had undetectable antibodies as indicated by the broken line. The level of receptor antibodies binding to the conformationally correct TSHR on CHO cells was highly variable in the immunized group.

### Antibody Responses to Hinge Region Epitopes

**Figure 5:**
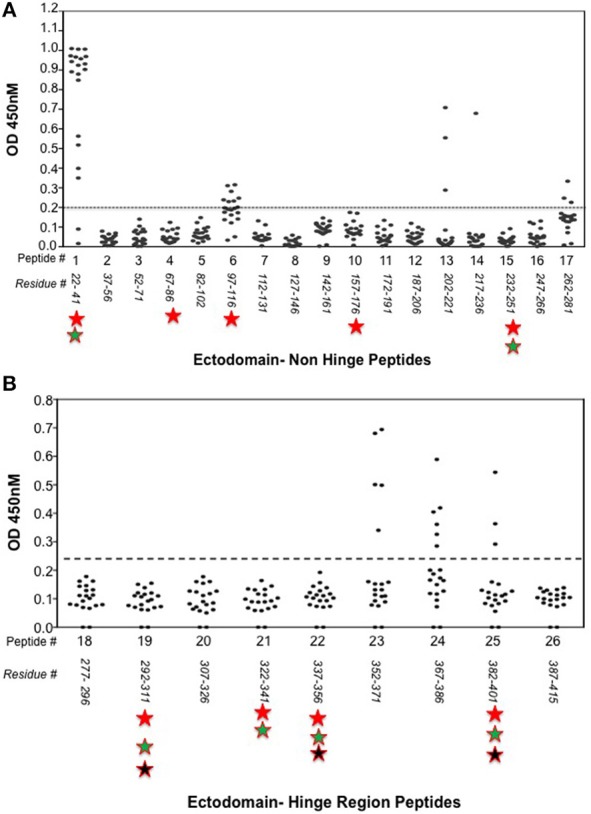
Detecting linear antibodies by indirect peptide ELISA. **(A)** Antibodies to non-hinge region peptides: 17 overlapping 20-mer peptides representing the non-hinge region of the TSHR ectodomain spanning residues 1–281 of the ectodomain was used in the indirect peptide ELISA to detect linear binding antibodies in the serum of immunized mice. The cut-off as indicated by the dotted line is the highest value of all peptides reacted with control animal serum plus 2 standard deviation above the mean. Peptide 1 (residues 22–41) of the receptor showed overwhelming predominance of antibody response (85%) compared to other peptides 6 and 13. Antigenicity predicted by more than two open source software is marked below each peptide by stars. **(B)** Antibodies to hinge region peptides: To examine hinge specific antibodies in the immunized animals we used 9 peptides covering the entire hinge region (residues 277–415). The majority of immunized animals showed linear binding antibodies to peptides 23, 24, and 25 that were above cut-off (dotted line) determined by the control serum samples plus 2 standard deviation. Overall, 40% of the immunized mice showed antibody response to these novel hinge regions “hot-spots.” Antigenicity predicted by more than two open source software is marked below each peptide by stars.

**Figure 6:**
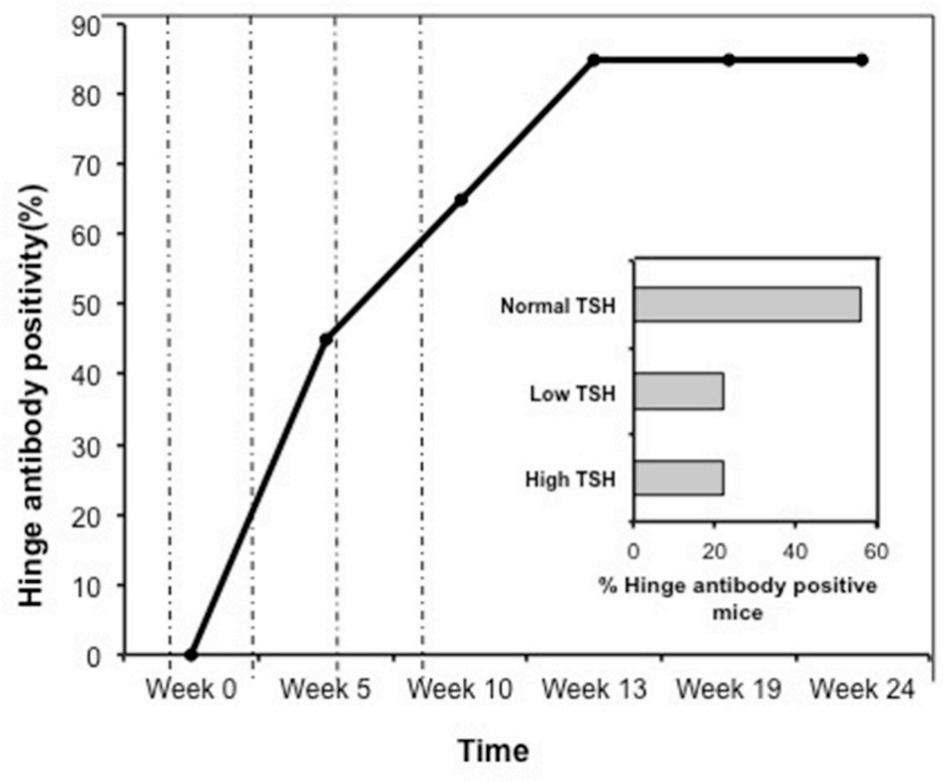
Time course of response to hinge region “hot-spots” in immunize mice. Mice immunized with full-length hTSHR showed increasing antibody response to the hinge region “hot-spots” throughout the time course of the immunization described in Figure 1. As indicated here an antibody rise started as early as < 5 weeks and plateaued off at week 13 maintaining 85% positivity until the termination of the experiment. Dotted lines indicate immunization time points. Insert: Shows the percentage of hinge region “hot-spot” antibody positive mice by thyroid function at week 10. 56% of the antibody positive mice were in the normal TSH range and 22% were in each of the low and high TSH ranges.

Indirect peptide ELISA's were performed with 17 of the 20-mer overlapping peptides covering the entire TSHR ECD and 9 peptides covering the entire TSHR hinge region. Positivity was determined using the cut-off criteria of ±2 *SD* above or below that of the control mice sera. Only 3 ECD peptides induced linear antibody responses with the major epitope residing at the N-terminus (aa 22–41) (Figure 5A). However, with the hinge region peptides the response was more heterogeneous, the data showed that 95% of the TSHR immunized animals showed antibody responses to at least one of the hinge region peptides (data not shown). Further, 70% of the immunized mice showed antibody responses which were restricted to three major peptides (Figure 5B) that we have termed hinge region “hot spot” epitopes (Figure 2). ELISA results from week 10 showed 25% of the receptor immunized animals had antibody responses to peptide 23 (residues 352–371), 30% to peptide 24 (residues 367–386), and 15% to peptide 25 (residues 382–401). The average of the difference in OD between the test animals and the cutoff ranged from 0.21 ± 0.09 to 0.80 ± 0.60 at 1:100 serum dilutions. However, the predicted antigenicity did not always correspond with the *in-vivo* responses against these peptides as indicated by stars under each epitope (Figure 5B). TSHR hinge region antibodies were detected as early as week 5 post-immunization in the test animals with the immune response tapering off at week 13 but sustained until week 24 (Figure 6). Overall the hinge antibodies remained detectable until the termination of the experiment. There was no significant difference observed in the hinge antibody response between the low and high TSH subgroups (Figure 6 inset).

### Thyroid Pathology

**Figure 7:**
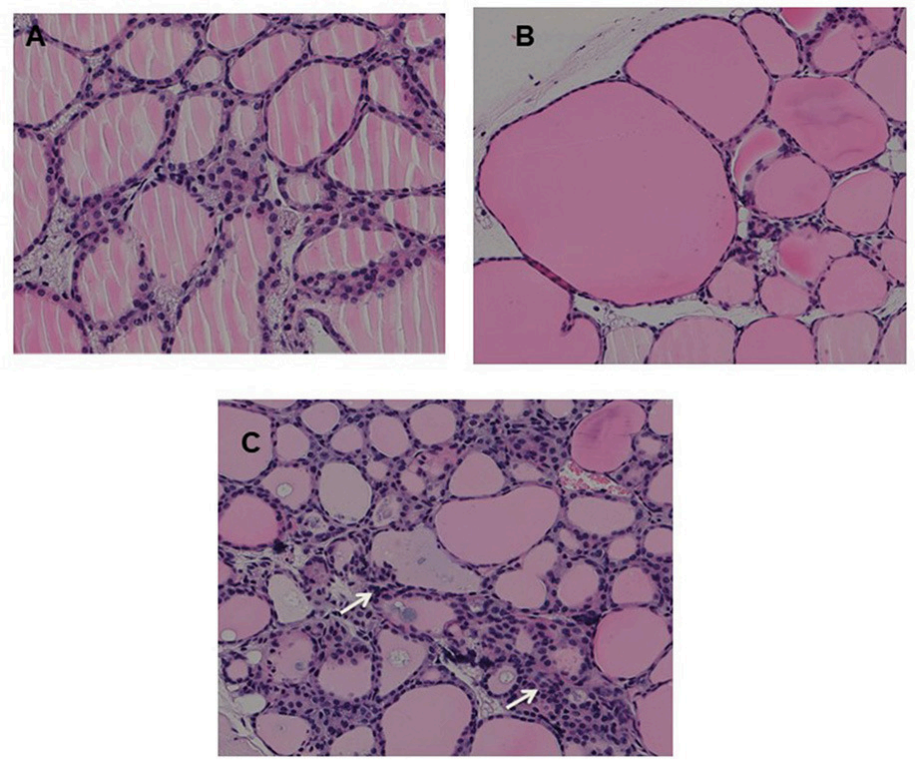
Thyroid histology of immunized animals: Images of H&E stained slides were obtained using the Hamamatsu NanoZoomer digital scanner at 20x magnification and analyzed and depicted here by NDP.view2 software at 15x magnification. **(A)** Normal control mouse thyroid **(B)** Enlarged thyroid follicles **(C)** Dense micro infiltrates. These changes marked by arrow were all seen in some high hinge positive mice (2 out of 5 hTSHR immunized mice). The changes could not be conclusively associated with these antibodies because of the heterogeneity of the response observed in these immunized animals.

Fixed thyroid sections of the TSHR immunized and control plasmid immunized mice were examined at 24 weeks for histological changes after H&E staining. Histological examination of thyroids from animals with antibodies directed to the hinge hotspots, in addition to other TSHR-Abs, showed variable pathological changes. These changes included enlarged thyroid follicles (Figure 7B) and mononuclear micro-infiltrates (Figure 7C). No such pathological changes were found in the control mice (Figure 7A). However, no correlation was observed between specific histological changes with levels of hinge region antibodies or thyroid function in the animals examined.

### Orbital pathology

**Figure 8:**
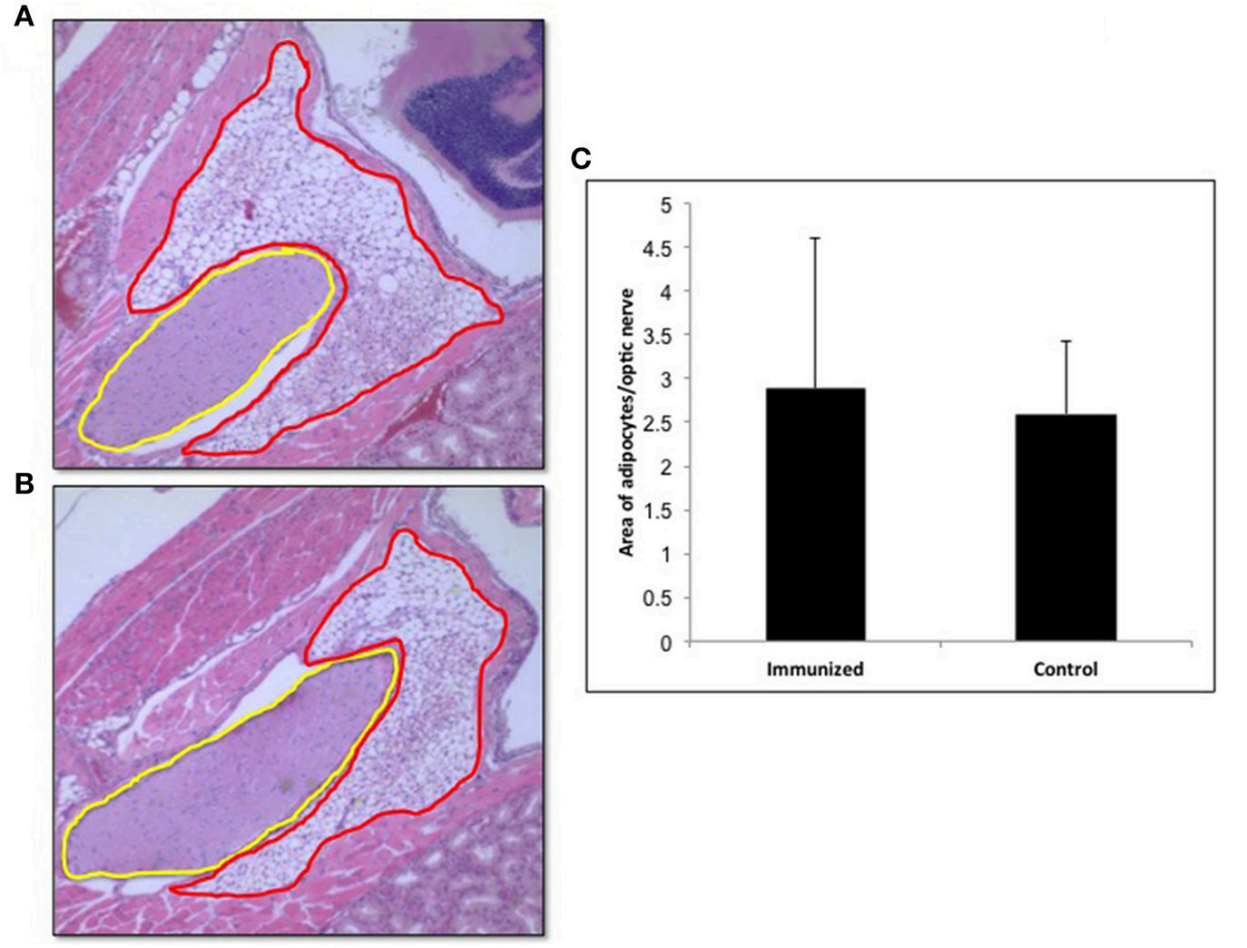
Adipogenesis in the retro orbital of immunized animals: Images of hematoxylin and eosin (H&E) stained slides were obtained using the Hamamatsu NanoZoomer digital scanner at 20x magnification and analyzed and depicted here by NDP.view2 software. Representative images of **(A)** Adipose tissue (ROI marked in red) around the optic nerve (ROI marked in yellow) of a control mouse. **(B)** Adipose tissue around the optic nerve of the TSHR immunized mice **(C)** To quantitate the changes in adipocytes in the control and immunized animals we used Image J on images at 10x magnification. The area of adipose was normalized by the area of the optical nerve on the same image for each orbit of animal. The immunized mice adipocyte to optic nerve area ratio was 2.88 ± 3.46 and that for the control mice was 2.59 ± 1.66. No significant difference in adipogenesis was seen in these immunized animals.

In view of recent reports that intensive TSHR-ECD immunization induced retro orbital adipocyte proliferation (26, 27), we examined several of the control (*n* = 5) and test (*n* = 10) mice after 24 weeks in order to detect periorbital adipose changes. Adipose tissue in the retro-orbital region was quantified using ImageJ and normalized to the area of optic nerve section. Quantitatively we did not observe any significant difference in the amount of adipose tissue between the immunized and control animals (Figure 8).

## Discussion

A full-length TSHR plasmid cDNA muscle immunization model was used to characterize the antigenicity of the hinge region of the TSHR ECD to reveal novel antigenic “hot spots” within the hinge region of the human TSHR. The full-length TSHR was used as the immunogen since that is what is produced endogenously by the thyroid cell prior to posttranslational processing although only a limited percentage of receptors may retain this complete structure at any one time because of subsequent receptor dimerization, cleavage, and ECD shedding (23, 28, 29).

TSHR autoantibodies are hallmarks for human autoimmune thyroid disease (AITD) which encompasses both Graves' disease and Hashimoto's thyroiditis (20, 30), but are heterogeneous in function. These antibodies can be broadly subdivided into three types; stimulating, blocking, and neutral, based on their behavior in functional TSH bioassays (3, 31). Autoantibodies of the stimulating, blocking, and neutral varieties also develop in TSHR immunization models (32, 33) so it is likely that the native tripartite structure of the TSHR (ECD, hinge, and TMD) helps define its antigenic character and repertoire of autoantibodies in animal models and patients with AITD. The development of stimulating monoclonal antibodies (34–36) along with the crystallization of human stimulating and blocking antibodies bound to a partial TSHR ECD (8) together with antibody protection analysis (10) have all provided deeper insight into the binding epitopes and mode of action of TSHR antibodies (8, 9). The large ECD consisting of residues 1–280 is a major pocket for TSH, stimulating and blocking antibody binding (7, 37). However, the remaining portion of the ECD from residues 280–410 defined as the “hinge region” (13, 16) was previously thought to be just a scaffold to transmit conformational changes to the TMD. However, later studies showed that the hinge was also a fertile region for TSH and autoantibody binding (16, 17). A detailed analysis of the various antigenic sites on the hinge region, which makes up more than a third of the ECD structure, has been lacking although there are several potential linear antigenic epitopes in this large segment of the TSHR. Studies from our laboratory, and by others (30, 32), have shown that the hinge region—including the cleaved sequence (residues 316–366)—is the region recognized by a class of antibodies often termed “neutral” antibodies (32) both in animal models (34) and in sera from GD patients (18, 38). These neutral antibodies recognize linear epitopes under fixed and unfixed conditions in flow-cytometry assays and they bind to their target antigen under reduced conditions in immunoblots.

Induced animal models for Graves' disease have been successfully developed with a variety of inbred and outbred rodents either using conformational TSHR antigen by injecting fibroblast cells expressing the TSHR (39) or immunization with TSHR-cDNA (40, 41) and especially successfully with adenoviral vectors incorporating the TSHR cDNA (42) or the ECD $\overset{]}{\underset{|}{\alpha }}$ (or A) subunit truncated at 289 (43). Although these models have provided unique insight into various aspects of GD pathogenesis by inducing hyperthyroidism via the production of stimulating TSHR antibodies they have not been able to provide an ideal GD model with associated Graves' orbitopathy (GO) and skin infiltration (called pre-tibial myxedema). Recently, a mouse model of GD was described with changes reminiscent of Graves' orbitopathy after using receptor plasmid DNA muscle immunization with the TSHR A subunit by square-wave electroporation (22). We used this approach with a full-length human TSHR plasmid construct and established that this immunization technique was able to generate receptor antibodies in 95% of the receptor immunized mice when assayed by flow-cytometry (Figure 4A) suggesting that the full-length receptor is highly antigenic. However, we observed that only a small fraction of the animals developed hyperthyroidism as determined by changes in their T4 and TSH level (Figures 3A,B) with none of the sera having detectable stimulating antibodies presumably due to very low levels or lacking functionality with the human TSHR rather than the mouse TSHR which we did not explore. Using a TSHR A subunit construct and the same immunization protocol, one recent study (44) observed that 75% of the immunized mice were hyperthyroid while another study found only a few 12.5% hyperthyroid and neither study measured stimulating TSHR-Abs directly (22) suggesting considerable variability in responses observed with same antigen.

Our laboratory has been studying neutral antibodies directed at the hinge region of the TSHR and has shown that despite being called “neutral,” they are capable of initiating multiple signaling cascades that lead to cell stress and programmed cell death of thyrocytes *in vitro* (20, 21) and *in-vivo*. Neutral antibodies bind to known linear epitopes within the hinge region of the ectodomain, especially the cleavage region, and binding of such antibodies could modulate TSHR post translational modifications including a decrease in receptor cleavage (45) and the induction of cell stress. Furthermore, the presence of neutral antibodies in Graves' patients (18) would suggest that these are not mere bystanders in the pathogenesis of the disease. Screening the entire hinge region for antibodies from our full-length TSHR immunized mice by peptide ELISAs identified three “hot spots” spanning amino acids 352–401 which related to peptides 23, 24, and 25 (Figures 2, 5B) in the hinge region. The heterogeneous responses observed in this inbred model is surprisingly similar to humans with AITD in which exposure to the same antigen may lead to different antibody responses and different clinical phenotypes. One of the “hot spot” peptide sequences contains a tyrosine (aa 385) for which sulfation has been shown to be important for activation of the receptor (11, 46). This would suggest that hinge antibodies directed specifically to this epitope might be important in modulating TSHR function and it has previously been observed (13, 47) that truncations encompassing the hinge region can increase the constitutive activity of the receptor confirming that it certainly is not an inert region.

While thyroid pathology was difficult to correlate with TSHR antibody responses we made a serious effort to observe changes in retroorbital adipogenesis around the optic nerve of the immunized mice compared to controls as reported by others (22, 44). However, we found no differences in the present study and this may have been due to the low level of stimulating TSHR antibodies generated by our construct (Figure 8) and this reported GO model requires further confirmation.

In summary, we used an intense muscle immunization mouse model of GD with the full length human TSHR in order to study the immune responses to the hinge region of the TSHR and found novel antigenic regions within the hinge. Our earlier studies have implicated that such neutral antibodies induce thyroid cell stress and apoptosis and we now demonstrate that a battery of hinge region antibodies are unavoidably produced in the immune response to the native full-length TSHR. However, their role in immunopathogenesis requires further evaluation. In this regard we have recently observed that treating mice with a monoclonal neutral TSHR–Ab induced thyroid pathology supporting our hypothesis that such antibodies influence the disease phenotype (48).

## Author Contributions

SiS conducted experiments, analyzed data, and wrote the manuscript. SaS design and data analysis. OS conducted experiment and analyzed data. SM contributed in data analysis. RM contributed in data analysis and discussion. RL designed and conducted experiments, analyzed data, and wrote the manuscript. TD contributed in data analysis and manuscript writing.

### Conflict of Interest Statement

The authors declare that the research was conducted in the absence of any commercial or financial relationships that could be construed as a potential conflict of interest.

## Abbreviations

TSHR: Thyroid stimulating hormone receptor
LRD: leucine rich domain
GD: Graves’ Disease
ECD: Ectodomain
TMD: Transmembrane domain
GPCR: G protein coupled receptor.
